# Vitamin D deficiency in Scheuermann’s disease is associated with increased adverse outcomes

**DOI:** 10.1051/sicotj/2024010

**Published:** 2024-04-03

**Authors:** Shivan N. Chokshi, Srikanth Mudiganty, Rutledge Carter Clement, William Accousti

**Affiliations:** 1 John Sealy School of Medicine 301 University Blvd, The University of Texas Medical Branch Galveston TX 77555 USA; 2 Department of Orthopaedic Surgery, Louisiana State University New Orleans LA 70112 USA

**Keywords:** Adverse outcomes, Hyperkyphosis, Kyphosis, Scheuermann’s disease, Vitamin D deficiency

## Abstract

*Introduction*: Scheuermann’s disease is a diagnosis of hyperkyphosis commonly encountered in pediatric patients. Studies in animal models suggest an association with vitamin D deficiency, however, extensive studies have not been performed in humans. This study analyzes the role of vitamin D deficiency on unfavorable results in patients with Scheuermann’s disease. *Methods*: The TriNetX database was utilized to perform a retrospective analysis. Patients in the United States aged 0–18 years with Scheuermann’s disease were identified using International Classification of Diseases, Tenth Revision (ICD-10) codes and categorized into those with and without a diagnosis of vitamin D deficiency. Comparison of patient groups depending on age, sex, ethnic origin, prior diagnosis of fibromyalgia, anxiety disorder, myositis, and major depressive disorder. Statistical analysis was conducted to identify the association between vitamin D levels and unfavorable results including pain, depression, suicide attempt, emergency department (ED) consult, hospitalization, and procedures on the spine or spinal cord. *Results*: In total, 11,277 patients were identified, 39% of whom had a concurrent diagnosis of scoliosis. A total of 1,024 (9.08%) were deficient in vitamin D. Patients with vitamin D deficiency had greater odds of pain (*P* < 0.0001), depression (*P* < 0.0001), suicide attempt (*P* = 0.0021), ED visits (*P* = 0.0246), and hospital admission (*P* < 0.0015). Conversely, patients with vitamin D deficiency had decreased odds of surgery on the spine or spinal cord (*P* = 0.0009). *Conclusion*: Vitamin D deficiency is associated with an elevated risk of pain, depression, suicide attempts, ED visits, and hospitalization. Our analysis highlights the need for more research to study the effect of vitamin D on Scheuermann’s disease.

*Level of evidence*: Level III, Prognostic

## Introduction

Scheuermann’s kyphosis (aka juvenile kyphosis), a subset of Scheuermann’s disease, is typically a structural kyphotic deformity of the thoracic spine identified by wedging of the anterior vertebral body, vertebral endplate irregularity, spinal stiffness, and backache near the apex of the deformity [1]. The pathology is seen in children and represents the second most common deformity of the growing spine after the various forms of scoliosis. To add further complexity, Scheuermann’s disease is often seen in concomitance with scoliosis in adolescence [2].

Despite these features prevalent in nearly 8% of the adolescent population in North America [3], there is a dearth of literature with regard to the factors leading to the disease. Although the true cause of Scheuermann’s disease remains unclear, current theories include growth irregularities, mechanical factors, genetic factors, and/or poor bone quality [4]. From a treatment perspective, bracing appears to be effective if a diagnosis is made prior to the curvature exceeding 50°–55° in actively growing patients. Surgical treatment may be indicated in patients with severe (>75°–80°) kyphosis with curve progression, refractory pain, or a neurological deficit [5].

Particularly in a disease that predominantly affects younger populations, there is a need to discover and implement conservative treatments to avoid significant patient discomfort or surgical intervention. Physical therapy is scarcely mentioned in the literature and there is little evidence that it can alter disease progression or improve long-term patient outcomes [6]. Although some research implied a correlation with low vitamin D levels, this has not yet been incorporated into the diagnosis and treatment framework for the disease and an extensive study has not been conducted that investigates whether vitamin D deficiency worsens patient outcomes [7].

Thus, the current analysis aimed to investigate the association between lack of vitamin D and unfavorable results in pediatric patients with Scheuermann’s disease, with the hypothesis that patients with Scheuermann’s disease and a vitamin D deficiency would have a higher rate of adverse outcomes studied.

## Methods

A retrospective cohort study was performed using the TriNetX multi-institutional research network. Institutional Review Board approval was not required as the platform only uses aggregated counts and summaries of de-identified patient information with no access to protected health information or personal data.

We queried the TriNetX Research Network using International Classification of Diseases, Tenth Revision (ICD-10) and Current Procedural Terminology (CPT) codes for patients aged 0–18 who were diagnosed with Scheuermann’s disease (ICD-10: M42.0) between January 1, 2018 and May 1, 2023. These patients were subsequently divided into 2 cohorts: Cohort 1 – patients with vitamin D deficiency, and Cohort 2 – patients with no vitamin D deficiency. Patients diagnosed with vitamin D insufficiency or vitamin D lab values in the insufficiency range were excluded from the analysis.

Prior to the final data analysis, we adjusted for confounding factors by integrating propensity score matching (PSM) from the TriNetX platform. Logistic regression was adopted to generate propensity scores and then applied “greedy nearest neighbor matching” with a caliper of 0.1 pooled standard deviations. Our cohorts were matched based on a 1:1 proportion on age, sex, ethnic origin, prior diagnosis of fibromyalgia, obesity, myositis, and anxiety. Statistical significance was defined as a two-sided alpha value <0.05.

The outcomes – pain, depression, suicide attempt, ED consult, hospitalization, and surgery on the spine or spinal cord were compared using odds ratios and 95% confidence intervals (95% CI). Independent *t*-test for continuous data and chi-square (χ^2^) test for categorical data were utilized with all tests two-tailed with an *α* level of 0.05. For the matched cohorts, multivariable conditional logistic regression analysis was computed to determine association under the assumption that exposures were independent of each other.

## Results

The TriNetX search identified a total of 11,277 Scheuermann’s disease patients aged 0–18 in the United States, of whom 64% were male and 36% were female. Thirty-nine percent of these patients had a concurrent diagnosis of scoliosis. Demographic information for patients with Scheuermann’s disease is shown in Table 1.

**Table 1:** Study demographics of Scheuermann’s disease patients. *N* = 11,277^a^.

Age (SD)	15.7 ± 3
Male (%)	7,217 (64)
Female (%)	4,060 (36)
White (%)	2,706 (24)
Black (%)	113 (1)
Asian (%)	0 (0)
American Indian or Alaska Native	0 (0)
Native Hawaiian or other Pacific Islander	0 (0)
Unknown race	8,458 (75)
Hispanic or Latino	225 (2)
Not Hispanic or Latino	2,819 (25)
Unknown ethnicity	8,232 (73)
Scoliosis	4,398 (39)
Dorsalgia	7,894 (70)
Fibromyalgia	564 (5)
Obesity	2,368 (21)

a Data are listed as mean ± SD or percentage (%). SD: standard deviation.

Of the 11,277 patients with Scheuermann’s disease, 1024 (9.08%) were identified as vitamin D deficient; however, only 1421 (12.6%) were tested for vitamin D levels. Thus, of those tested, 72% were found to be deficient. Propensity matching resulted in 1012 patients in both the vitamin D-deficient and normal vitamin D cohorts (Table 2). Table 3 shows the odds-ratio estimates derived from the analysis for adverse events. Children with insufficient vitamin D levels had 1.79 (95% CI 1.36–2.37, *P* < 0.0001) times the odds of pain, 2.09 (95% CI 1.81–2.42, *P* < 0.0001) times the risk of depression, 2.88 (95% CI 1.42–5.83, *P* = 0.0021) times suicide attempt risk, 1.13 (95% CI 1.01–1.28, *P* = 0.0246) times the incidence of ED visits, and 1.38 (95% CI 1.13–1.69, *P* < 0.0015) times the risk of hospitalization. Conversely, children with low Vitamin D levels had 0.53 (95% CI 0.37–0.78, *P* = 0.0009) times the odds of surgical procedures on the spine or spinal cord.

**Table 2:** Propensity Score Matching for Scheuermann’s Disease and Vitamin D Deficiency^a^.

Demographic	Vitamin D deficiency (original) *n* = 1024	No Vitamin D deficiency (original) *n* = 10,253	SMD before matching	Vitamin D deficiency (matched) *n* = 1012	No Vitamin D deficiency (matched) *n* = 1012	SMD after matching	*P* value after matching
Age (SD)	16.6 ± 5.5	14 ± 4.84	0.5129	15.5 ± 5.47	15.3 ± 5.22	0.0558	0.2098
Male (%)	556 (54.297)	6,650 (64.91)	0.2176	556 (54.941)	545 (53.854)	0.0218	0.6235
Female (%)	468 (45.703)	3,594 (35.081)	0.2178	456 (45.059)	467 (46.146)	0.0218	0.6235
White (%)	281 (27.441)	2,367 (23.104)	0.0999	278 (27.47)	281 (27.767)	0.0066	0.8814
Black or African American (%)	12 (1.172)	98 (0.957)	0.021	12 (1.186)	14 (1.383)	0.0176	0.693
Asian (%)	10 (0.977)	10 (0.098)	0.1205	10 (0.988)	0 (0)	0.1413	0.0015
American Indian or Alaska Native (%)	0 (0)	0 (0)	–	0 (0)	0 (0)	–	–
Native Hawaiian or other Pacific Islander (%)	0 (0)	0 (0)	–	0 (0)	0 (0)	–	–
Unknown race (%)	729 (71.191)	7,772 (75.861)	0.106	720 (71.146)	717 (70.85)	0.0065	0.8832
Hispanic or Latino (%)	38 (3.711)	217 (2.118)	0.0948	38 (3.755)	39 (3.854)	0.0052	0.9075
Not Hispanic or Latino (%)	304 (29.688)	2460 (24.012)	0.1283	300 (29.644)	297 (29.348)	0.0065	0.8837
Unknown ethnicity (%)	682 (66.602)	7568 (73.87)	0.1595	674 (66.601)	676 (66.798)	0.0042	0.9248
Anxiety disorder	325 (31.738)	1117 (10.903)	0.526	314 (31.028)	319 (31.522)	0.0107	0.8105
Obesity	329 (32.129)	1107 (10.805)	0.5378	319 (31.522)	343 (33.893)	0.0506	0.2555
Fibromyalgia	91 (8.887)	229 (2.235)	0.2934	84 (8.3)	79 (7.806)	0.0182	0.683
Myositis	90 (8.789)	221 (2.157)	0.2947	84 (8.3)	74 (7.312)	0.0368	0.4074

a Data are listed as mean ± SD or percentage (%). SMD: standard mean difference.

**Table 3:** Risk analysis of adverse outcomes in matched patients with Scheuermann’s Disease^a^.

	Vitamin D deficiency		No vitamin D deficiency				
Outcome	*n*	%	*n*	%	*P*	OR	Odds CI
Pain	61	6.055	35	3.465	<0.0001*	1.795	1.36–2.37
Major depressive disorder	293	29.004	165	16.32	<0.0001*	2.095	1.812–2.421
Suicide attempt	10	0.977	3	0.342	0.0021*	2.877	1.42–5.827
Emergency department visit	413	40.82	90	37.98	0.0246*	1.126	1.011–1.284
Hospital admission	120	11.914	81	8.912	0.0015*	1.382	1.131–1.69
Surgical procedures	45	4.447	81	8.004	0.0009*	0.535	0.367–0.779

a CI: confidence interval, OR: odds ratio, *P*: *p*-value.

A subgroup analysis was performed analyzing the odds of the same outcomes in only those patients with a recorded Vitamin D level. Table 4 shows the odds-ratio-derived estimates from the subgroup analysis for adverse events. Patients with a diagnosed vitamin D deficiency and recorded vitamin D levels confirming the deficit had worsened the risk of pain, depression, suicide attempt, ED visits, and hospitalization. Thus, the subgroup analysis did not suggest a discrepancy in outcomes between patients with a diagnosis of vitamin D deficiency and patients without a diagnosis of vitamin D deficiency and recorded vitamin D levels.

**Table 4:** Subgroup analysis of adverse outcomes in matched patients with Scheuermann’s disease and recorded vitamin D levels^a^.

	Vitamin D deficiency		No vitamin D deficiency				
Outcome	*n*	%	*n*	%	*P*	OR	Odds CI
Pain	74	7.21	36	3.465	<0.0001*	2.106	2.016–2.199
Major depressive disorder	278	27.13	149	16.32	<0.0001*	2.186	2.139–2.235
Suicide attempt	11	1.048	3	0.342	<0.0001*	2.966	2.611–3.369
Emergency department visit	402	39.311	342	37.98	0.0246*	1.291	1.270–1.313
Hospital admission	126	12.359	84	8.912	0.0011*	1.567	1.519–1.616
Surgical procedures	32	3.147	63	8.004	0.008*	0.489	0.464–0.514

a CI: confidence interval, OR: odds ratio, *P*: *p*-value.

## Discussion

Vitamin D deficiency has been described as having adverse effects on the general well-being of an individual [8], but its role in Scheuermann’s disease is still largely unexplored. The results of our data analysis suggest a strong correlation between vitamin D deficiency and the risk of adverse events in patients with Scheuermann’s disease. Specifically, patients with vitamin D deficiency had worse pain, depression, unsuccessful suicide attempts, hospital admissions, and ED visits. However, patients with vitamin D deficiency had decreased odds of spinal and spinal cord surgical procedures. The greatest association was seen in suicide attempts, in which vitamin D-deficient patients were nearly three times more likely when compared to patients who did not have a vitamin D deficiency. An important consideration is that only a small fraction (12.6%) of patients was tested for vitamin D levels. Despite this, a large majority (72%) of those tested were diagnosed with vitamin D deficiency. Thus, the data presented is not a summary of all children with Scheuermann’s disease in the dataset with vitamin D deficiency, but rather just the ones that were identified by testing. This may suggest that the true number of vitamin D deficient patients with Scheuermann’s disease is likely higher than the number of patients observed in the data due to low testing rates. Although there is no literature precedent for the results obtained because of a lack of risk factor analysis and vitamin D investigations in patients with Scheuermann’s disease, there is still a basis of validity for our results.

This study has limitations. Given the retrospective nature of this database study, associations of the variables could be determined without necessarily achieving causation, even though care was given to the creation of the cohorts to minimize this effect. In addition, because the patients were identified by the use of CPT codes, it is likely that not all patients of the target population were captured if there was a failure to enter the patient’s CPT-associated diagnosis into the clinical database, especially if it is a secondary diagnosis (i.e., vitamin D deficiency or insufficiency). Thus, there are segments of data in each portion of the analysis that are invariably missing. Lastly, the inability to tightly control patient information and additional comorbidities means there is a possibility of confounding variables that were not accounted for. Cohorts were balanced based on important differentiating factors; however, not all comorbidities can be captured either due to database limitation or lack of clinical entry.

Vitamin D plays an important role in maintaining a healthy mineralized skeleton [9]. Vitamin D is essential to the regulation of calcium and phosphorus levels, which maintain essential cellular functions and promote bone mineralization [10]. Thus, vitamin D insufficiency and deficiency are now recognized as major causes of metabolic bone disease [9]. Hypovitaminosis D adversely affects calcium metabolism, osteoblastic activity, matrix ossification, bone remodeling, and bone density. In the developing skeleton, this is a cause of rickets and poor bone health [6]. While specific associations between vitamin D deficiency and kyphotic changes have not yet been described in the literature, analysis of porcine spines has shown that a maternal diet deficient in vitamin D increases the development of hyperkyphosis in offspring [7]. This, in addition to known information regarding the role of vitamin D on bone health, may suggest a relationship between vitamin D levels and kyphosis in the developing spine.

Our findings indicate that vitamin D deficiency may augment important and devastating sequelae of Scheuermann’s disease in children. A survey of the literature suggests that depression and suicide have not been studied in depth in pediatric patients with disease and deformity of the spinal column; however, there are some with which our results can be related. Klaas et al. [11] found, in their analysis of pediatric patients with spinal cord injury, that there was a higher risk of anxiety and depression with a positive correlation seen with age. Further studies in both Taiwan and the United States have found that adolescent idiopathic scoliosis (AIS) was associated with an increased risk of depression, potentially due to increased pain and insomnia [12–14]. Thus, even though suicidality has not been well-described in the orthopedic literature, there is a basis for preliminary understanding that AIS can be associated with higher levels of depression. The current literature, in tandem with the results obtained in this data analysis, should prompt healthcare professionals to consider designing and planning effective psychological attention in the treatment of Scheuermann’s disease.

The conclusions derived from this data should serve to set the stage for further investigations into Scheuermann’s disease and its association with vitamin D deficiency. The data presented suggests remarkable differences in a myriad of adverse outcomes or co-morbidities in patients with vitamin D deficiency, including pain, depression, suicide attempts, hospital admission, and ED visits. Consequently, it is essential to understand the involvement of vitamin D in Scheuermann’s disease, including more consistent guidelines for checking vitamin D levels and a fresh look at conservative strategies for patient treatment.

## Funding

This research did not receive any specific grant from funding agencies in the public, commercial, or not-for-profit sectors.

## Conflicts of Interest

Authors declare no conflict of interest.

## Data availability statement

Data will be made available on request.

## Author contribution statement

Institutional Review Board approval was not required as the platform only uses aggregated counts and summaries of de-identified patient information with no access to protected health information or personal data.

## Ethics approval

S.N. Chokshi: Investigation, Data collection, Formal analysis, Writing original draft, Writing – review & editing. S. Mudiganty: Writing – original draft, Writing – review & editing, submission. R.C. Clement III: Methodology, Supervision, Writing – review & editing. W. Accousti: Conceptualization, Methodology, Supervision, Writing review & editing.
